# Spanish cross-cultural adaptation and validation of the Australian Pelvic Floor Questionnaire in running women

**DOI:** 10.1038/s41598-022-12043-5

**Published:** 2022-05-18

**Authors:** Guadalupe Molina-Torres, Marina Guallar-Bouloc, Alejandro Galán-Mercant, Martyna Kasper-Jędrzejewska, José Antonio Merchán-Baeza, Manuel Gonzalez-Sanchez

**Affiliations:** 1 Department of Nursing, Physiotherapy and Medicine, Faculty of Health Sciences, University of Almería, 04120 Almería, Spain; 2 Department of Physiotherapy, Faculty of Health Science, University of Jaén, 23071 Jaén, Spain; 3 Biomedical Research and Innovation Institute of Cádiz (INiBICA) Research Unit, Puerta del Mar University Hospital, University of Cádiz, 11002 Cádiz, Spain; 4 MOVE-IT Research Group, Department of Physical Education, Faculty of Education, Sciences University of Cádiz, 11002 Cádiz, Spain; 5 Institute of Health Sciences, Opole University, Plac Kopernika 11a, 45-040 Opole, Poland; 6 Research Group on Methodology, Methods, Models and Outcomes of Health and Social Sciences (M3O), Faculty of Health Science and Welfare, University of Vic-Central University of Catalonia (UVIC-UCC), 08500 Vic, Spain; 7 Department of Physiotherapy, Faculty of Health Sciences, University of Málaga, 29071 Málaga, Spain; 8 Institute of Biomedicine of Málaga (IBIMA), 29010 Málaga, Spain; 9 Physiotherapy Area, Department of Nursing and Physiotherapy, Faculty of Nursing and Physiotherapy, University of Cádiz, C/CP, 11002 Cádiz, Spain

**Keywords:** Health care, Medical research, Signs and symptoms, Urology

## Abstract

Pelvic floor dysfunctions are a wide range of disorders in the gynaecological, lower urinary and gastrointestinal tracts that affect the structure and/or function of the pelvic organs. The objective of this study was to carry out a cross-cultural adaptation and a psychometric analysis of the Spanish version of the Australian Pelvic Floor Questionnaire. Observational study divided into two main phases: (1) translation and cross-cultural adaptation and (2) psychometric tests. Women runners from all over the Spanish territory, from different federations, clubs and levels were recruited. Participants: 424 female runners, native Spanish, over 18 years of age and who had been practicing running for more than 6 months. The instruments used in this study were the Australian Pelvic Floor Questionnaire, Female Sexual Function Index, King Health Questionnaire, Quality of Life SF-12 and EuroQoL 5-D. The Spanish version of Australian Pelvic Floor Questionnaire has proven to be an understandable and easy-to-use tool. The general internal consistency of the questionnaire was 0.972 and the intraclass correlation coefficient ranged between ICC 0.596–0.960. The Spanish version of Australian Pelvic Floor Questionnaire is a valid and reliable measure that can be used clinically to assess pelvic floor dysfunctions among the female Spanish population.

## Introduction

Pelvic floor dysfunctions (PFDs) are a wide range of disorders in the gynaecological, lower urinary and gastrointestinal tracts that affect the structure and/or function of the pelvic organs^1^, they are also very common after childbirth, with approximately 30% of mothers experiencing urinary incontinence (UI) and 10% anal incontinence (AI)^2^; moreover, they can also present pain and pelvic organ prolapse (POP)^3^, although it should be noted that UI is a health problem that affects the quality of life among women of all ages^4^. On the other hand, female sexual disorders must also be considered within PFDs, since they are alterations in the sensation and/or normal function experienced by a woman during sexual activity and can be classified as disorders of sexual interest/arousal, orgasmic disorder and pelvic-genital pain/penetration disorder^5^. PFDs cause discomfort and reduce the quality of life, including lower participation in physical activity and exercise^6^, with regular and progressive training of the pelvic floor muscles^7^ and biofeedback^8^ being the treatments of choice, among others.

The PFD are usually associated with events such as childbirth and menopause^9^, however, the increase in the practice of physical activity by the general female population has caused the incidence of UI to rise to 22.9% in young, active, nulliparous women^9^. And the incidence level to rise to 30.7% when we talk about runners and reach 45–60% when talking about the marathon distance^10,11^. In addition, there are other PFDs that have a higher level of incidence in female runners. Specifically, In the case of POP, 12.7% of female runners reporting having symptoms^9^, for AI, the prevalence is quite wide, from 35 to 60%^12^ , 34% of female recreational runners suffer from solid or liquid fecal leaks and gas continence problems. And if we focus only on gas control, the prevalence rises to 29.9%^9^. In addition, sexual dysfunctions in the general population reach 30–50% prevalence^10^. Similarly, a prevalence of dyspareunia (pain during sexual intercourse) of 20.1% has been described in women who practice intensive sports (+ 8 h of training/week or high level) and 9.4% in women who practice non-intensive sports^11^. We also found 57.6% of active women present sexual function problems^13^. For all of the above, female runners could be considered a specific risk group for PFD problems.

In the last two decades, the use of Patient-Reported Outcome Measures (PROM)^14^ has increased exponentially both in clinical and research environments, since they are economical, reliable and specific tools that allow evaluating subjective aspects that a patient may perceive as altered results of their pathology, such as quality of life, general health, disability, etc. PROMs allow clinicians, researchers and patients to interpret, in a simple way, the results of the evolution of the latter and the changes that occur in their symptoms, capacity and function^15^. Likewise, in recent years, the use of scales and questionnaires has been extended to assess aspects of pelvic floor dysfunction and its severity and impact on the quality of life^16,17^. Although these questionnaires are very useful, especially in research results, most of them do not cover all aspects of pelvic floor dysfunction: bladder, bowel, prolapse, and symptoms of sexual dysfunction. That is why the Australian Pelvic Floor Questionnaire (APFQ) should be highlighted, which evaluates all pelvic floor symptoms, including bladder, bowel, sexual function, prolapse symptoms, symptom severity, impact on the quality of life and discomfort in women with pelvic floor disorders^18^. Despite the widespread cross-cultural validation of the APFQ in pelvic floor dysfunction in other countries^19–22^, there is no version of the APFQ validated in Spanish, which is one of the five UN languages^23^ and the second most spoken native language in the world^24,25^. The adaptation of this tool to Spanish could enable the evaluation of pelvic floor dysfunctions in Spanish-speaking women, regardless of whether or not they perform sports with an impact on the pelvic floor; this would provide objective assessment tools and allow planning therapeutic strategies, for the prevention of pelvic floor dysfunctions and for their treatment once they have been established, which, in turn, can affect the sports performance and quality of life of these women. Consequently, the aim of this study was to carry out a cross-cultural adaptation and psychometric analysis of the Spanish version of the APFQ.

## Methods

### Study design

To carry out the cross-cultural adaptation and validation of the Australian Pelvic Floor Questionnaire (APFQ) into Spanish, an observational study divided into two main phases was developed: (1) translation and cross-cultural adaptation, and (2) psychometric tests.

### Participants

Women runners from all over the Spanish territory, from different federations, clubs and levels were recruited. The inclusion criteria were: (1) Spanish natives over 18 years of age, (2) female runners and (3) more than 6 months practicing running sports. On the other hand, the study excluded: (1) those participants who abandoned the study without answering any of the questions of a questionnaire were excluded, and (2) those who presented a cognitive impairment that did not allow them to understand and/or answer the forms.

### Ethical considerations

This study was developed following the recommendations of the Declaration of Helsinki in accordance with the ethical principles for research in human beings, and the data were used in accordance with Organic Law 3/2018, of December 5, on the Protection of Personal Data and guarantee of digital rights. All participants signed an informed consent to be part of the study. In addition, the Ethics Committee of a Spanish University approved the realisation of this study, with protocol number UVIC-CCC 81/2019.

### Australian Pelvic Floor Questionnaire

The text of the APFQ questionnaire used in this study consists of 42 questions about the symptoms of PFDs. It has four domains: bladder function (Q1–15), bowel function (Q16–27), prolapse symptoms (Q28–32), and sexual function (Q33–42). The resulting scores were divided by the number of relevant questions within each domain and multiplied by 10, giving a value between 0 and 10 for each of the four domains and an overall score of 40 for pelvic floor dysfunction. Cronbach's alpha for the four APFQ domains was: bladder function 0.72, bowel function 0.82, pelvic organ prolapse 0.95, and sexual function 0.81^18^.

### Translation and cross-cultural adaptation

To ensure terminological and conceptual equivalence, in the questions that make up the APFQ, the recommendations of the International Test Commission Guidelines for test translation and adaptation were followed^26^, as well as those of the World Health Organisation (WHO)^27^.

**Figure 1:**
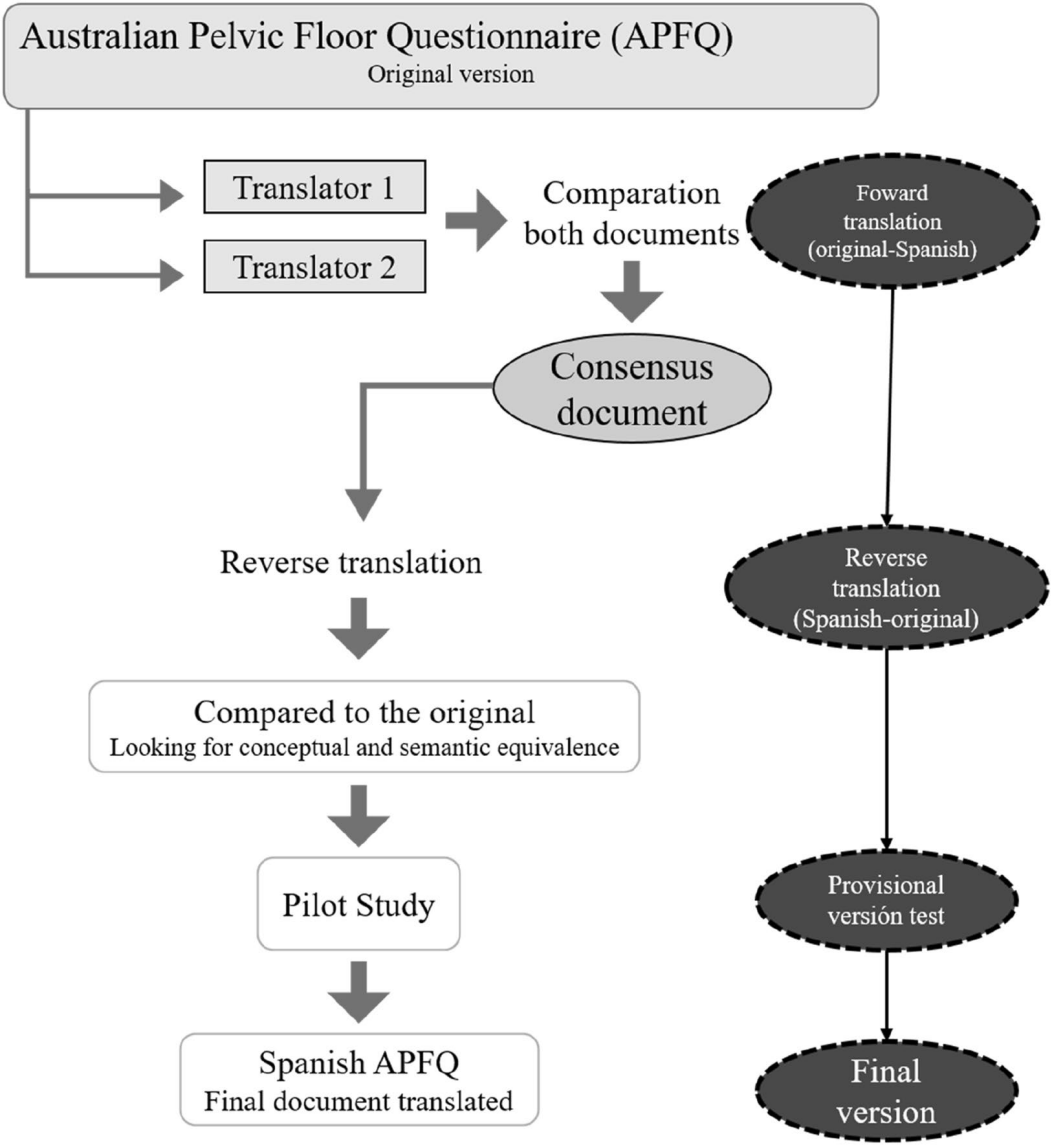
Flowchart of the development process APFQ Spanish version.

The process of the adaptation of the Spanish version of the APFQ from it English version can be broken down into a 5-step protocol: English to Spanish translation of the APFQ, performed by two independent and blinded native Spanish speakers; the two independent versions of the APFQ-Sp were compared and an agreement was reached to develop the preliminary version of the APFQ-Sp; subsequent back-translations (from Spanish to English) were done independently by two native English translators. Any discrepancies in the translation were discussed and resolved by a committee of 5 experts, obtaining a preliminary version. The preliminary version of the APFQ-Sp was subjected to a pilot test, with a sample of 25 participants (Fig. 1).

### Questionnaires used for construct validity

#### Female Sexual Function Index (FSFI)

The Female Sexual Function Index (FSFI) questionnaire is a self-administered instrument, consisting of 19 items that refer to the last 4 weeks. These items are grouped into six domains or subscales: sexual desire (items 1 and 2), arousal (items 3, 4, 5, 6), lubrication (items 7, 8, 9, 10), orgasm (items 11, 12, 13), satisfaction (items 14, 15, 16) and pain (items 17, 18, 19). The subscales range from 0 (or 1) to 5, and the sum of the scores of these six subscales yields an overall female sexual function score (with higher scores indicating better sexual function), with a Cronbach's alpha of 0.97 for the total score^28^. In a Spanish version tested in menopausal women, Cronbach's alpha for the total score was 0.964^29^, and in another Spanish version, Cronbach's alpha was 0.85 for the total score^16^.

#### King Health Questionnaire (KHQ)

The King Health Questionnaire measures the impact of urinary incontinence in the physical, social and mental areas, specifically to assess the quality of life in patients with urinary incontinence. It consists of 21 questions distributed in eight different dimensions, with a scale to measure the severity of urinary symptoms. The range of scores for each dimension goes from 0 (best quality of life) to 100 (worst quality of life), with a Cronbach's alpha above 0.72 in all domains^30^, and the one obtained in the Spanish version was 0.911^31^.

#### Quality of life SF-12

The SF-12v2 consists of a set of 12 items on health-related quality of life (HRQOL), which shows two reduced scores: on the one hand, the state of the physical component (PCS) and, on the other hand, the state of the mental component (MCS), on a scale of 0 to 100. It also features eight additional domains (physical functioning, physical role, bodily pain, general health, vitality, social functioning, emotional role, and mental health). Both additional domains and summary scores were calculated using algorithms where each item response has an individual weight in the total score. Higher scores indicate better perceived HRQoL^32^.

#### EuroQoL 5-D

EuroQol-5D is a questionnaire to measure people's quality of life. It is made up of 5 domains (mobility, self-care, regular activities, pain/discomfort, and anxiety/depression) divided into three severity levels (no problems, some problems or moderate problems, and serious problems). This system also includes a visual analog scale (EQ-5D VAS) defined by a vertical scale of 10 cm at each end, which are the extreme expressions of self-perceived health status ranging from 0 (worst health) to 100 (best health). Its reliability obtained a Cronbach's α = 0.53^33^.

### Data collection

All participants completed the following questionnaires: The Spanish version of the Australian Pelvic Floor Questionnaire (APFQ-Sp), Female Sexual Function Index (FSFI), King Health Questionnaire (KHQ), EuroQoL Quality of Life Questionnaire (5D and VAS), the questionnaire on the general state of health SF-12 (Short form-12) and sociodemographic information. Following the results published in previous studies^34,35^, in which higher levels of internal consistency and reliability are observed in periods of less than 7 days between the two measurements, the APFQ-Sp was filled twice with a difference of 3–5 days between measurements . The FSFI, KHQ and QoLSF-12 questionnaires were used to analyse the construct validity of the APFQ-Sp. The data were obtained between October 2020 and July 2021. Two blinded researchers external to the study performed the data collection, as well as the data analysis.

### Data analysis

A frequency analysis of some of the characteristics of the sample was performed, as well as a descriptive analysis of the sociodemographic variables, including the outcome measures used (APFQ-Sp, IFSF, KHQ, SF-12 and EuroQoL5D), calculating the mean and the standard deviation. To analyse the distribution and normality of the sample, the Kolmogorov–Smirnov test was used. Floor and ceiling effect were analysed.

The Cronbach's α coefficients were calculated to analyse the internal consistency of the measures. In addition, the response to the item was analysed using the Intraclass Correlation Index (ICC–2:1). The reliability values were classified according to the following scale: Poor: ≤ 0.40; Moderate: 0.40–0.60; Good: 0.60–0.80; Excellent: ≥ 0.80^36^.

The formula ${\text{SEM }} = \, s\sqrt {1 - r}$ was used to calculate the standard error of measurement (SEM). For both measures (APFQ-Sp1 and APFQ-Sp2) the test score's standard deviation was “s”, and “r” was Pearson's correlation coefficient. Following the analysis described by Stratford^37^, to measure the sensitivity of the tool, the minimal detectable change 90 (MDC90) was used. The formula used to calculate the MDC90 was as follows: MDC90 = SEM × √2 × 1.65. The floor or ceiling effect was considered to be present if more than 15% of the participants reached the lowest or highest score, respectively.

The structure and validity of the construct was analysed from the extraction by maximum likelihood (EMV). To maintain the original structure of the APFQ, a 4-factor forced model was performed. In addition, to perform the EMV, the requirement of a minimum of 10 subjects per item was satisfied (minimum number 420 – subjects measured 424)^38^.

Criterion validity was calculated by analysing the degree of correlation between the APFQ-Sp and the Spanish versions of the questionnaires: FSFI^16,29^, KHQ^31^, QoLSF-12^32^ and EuroQoL 5-D^33^. Pearson's correlation coefficient was structured according to the following scale: r ≤ 0.49 (poor), 0.50 ≤ r ≤ 0.74 (moderate), r ≥ 0.75 (strong)^39^.

To perform the statistical analysis of this study, the SPSS statistical treatment programme (V.23.0) was used.

## Results

### Translation and cross-cultural adaptation

The translated and culturally adapted version of the APFQ into Spanish (APFQ-Sp) is presented in Supplementary File S1. The Table 1 shows the anthropometric characteristics of the participants. The total of 424 women who participated in this study had a mean age of 38.56 (± 9.064) years. More than 75% of the participants had a university level of education (bachelor's, master's or doctorate). Almost half of the participants had not had a previous pregnancy. In addition to this, the type of delivery, number of previous abortions, etc., can be analysed in depth. On the other hand, the sports and federative data of the participants are also presented.

**Table 1:** Characteristics of the study population.

		Frequency	Percentage	Accumulated percentage
Educational level	Compulsory education	20	4.7	4.7
	Vocational training	64	15.1	19.8
	University studies	214	50.5	70.3
	Master	105	24.8	95.0
	Doctoral studies	21	5.0	100.0
Number of pregnancies	0	194	45.8	45.8
	1	63	14.9	60.6
	2	116	27.4	88.0
	3	34	8.0	96.0
	4	12	2.8	98.8
	5	3	0.7	99.5
	6	2	0.5	100.0
Number of vaginal deliveries	0	246	58.0	58.0
	1	67	15.8	73.8
	2	85	20.0	93.9
	3	21	5.0	98.8
	4	4	0.9	99.8
	10	1	0.2	100.0
Number vaginal deliveries suction cup	0	384	90.6	90.6
	1	37	8.7	99.3
	2	2	0.5	99.8
	9	1	0.2	100.0
Number forceps vaginal deliveries	0	392	92.5	92.5
	1	30	7.1	99.5
	2	2	0.5	100.0
Number of episiotomy deliveries	0	304	71.7	71.7
	1	71	16.7	88.4
	2	45	10.6	99.1
	3	4	0.9	100.0
Number of deliveries with tears	0	360	84.9	84.9
	1	53	12.5	97.4
	2	10	2.4	99.8
	3	1	0.2	100.0
Cesarean section	0	361	85.1	85.1
	1	37	8.7	93.9
	2	25	5.9	99.8
	3	1	0.2	100.0
Number of abortions	0	334	78.8	78.8
	1	61	14.4	93.2
	2	24	5.7	98.8
	3	4	0.9	99.8
	4	1	0.2	100.0
Federated	Yes	163	38.4	38.4
	No	261	61.6	100.0
Level of competition	Provincial	81	19.1	19.1
	Regional	76	17.9	37.0
	National	44	10.4	47.4
	International	17	4.0	51.4
	I don't do competitions	206	48.6	100.0
Practice of another sport activity	Yes	331	78.1	78.1
	No	93	21.9	100.0
Practice of another sport activity	None	88	20.8	20.8
	Pilates, abdominal work, yoga, stretching	16	3.8	24.5
	Functional training	21	5.0	29.5
	Impact sports	13	3.1	32.5
	Cardio sports	140	33.0	65.6
	Strength	44	10.4	75.9
	Combination of 2 or more	102	24.1	100.0
Specific pelvic floor work	Hypopressives	57	13.4	13.4
	Kegel exercises	23	5.4	18.9
	5P Method	4	0.9	19.8
	I do not do any specific work	285	67.2	87.0
	Other	12	2.8	89.9
	Hypopressives + Kegel	31	7.3	97.2
	Hypopressives + other	5	1.2	98.3
	Hypopressives + Kegel + 5P	4	0.9	99.3
	Kegel + 5P	2	0.5	99.8
	Kegel + other	1	0.2	100.0

Table 2 shows the mean, minimum, maximum and standard deviation values of all the assessment tools used in this study, that is, the Australian Pelvic Floor Questionnaire (APFQ-Sp), the Female Sexual Function Index (FSFI), King Health Questionnaire (KHQ), EuroQoL Quality of Life Questionnaire (5D and VAS), the questionnaire on the general state of health SF-12 (Short form-12). Moreover, the values of the different sub-indices or sections in which the different tools are divided are presented. When performing the floor effect and ceiling effect analysis, it was observed that 23 (0.54%) and 16 (0.37%) participants reached the minimum and maximum APFQ-Sp score, respectively. Given these results, the floor/ceiling effects were considered not relevant.

**Table 2:** Mean values and variance ranges of the questionnaires used for criterion validity.

		Minimum	Maximum	Mean	Standard deviation
Years		18	67	38.56	9.064
APFQ	Total	0	0.45	0.11	0.081
	Urinary tract section	0	0.56	0.12	0.109
	Defecatory section	0	0.79	0.15	0.111
	Prolapse section	0	0.80	0.03	0.093
	Sexual activity section	0	1.00	0.15	0.194
FSFI	Total	0	5	1.015	1.031
	Orgasm	0	11	1.097	1.275
	Satisfaction	0	11	1.028	1.211
	Pain	0	4	0.873	0.897
	Desire	1	5	3.07	1.023
	Excitement	0	5	3.60	1.563
	Lubrication	0	5	3.67	1.692
	General health perceptions	0	100	27.59	22.129
KHQ	Incontinence impact	0	100	31.67	18.627
	Part 1	25	200	59.269	31.217
	Role limitation	0	150	37.866	26.624
	Physical Limitation	0	200	57.618	29.154
	Social limitation	0	125	34.611	22.946
	Personal Relationships	75	275	79.658	19.910
	Emotions	75	300	88.090	33.681
	Sleep energy	50	200	58.255	20.692
	Severity	100	325	129.776	43.378
	Part 2	450	1250	529.422	123.781
	Part 3	0.00	650	80.366	95.460
SF-12	Physical Function	22.11	67.16	49.271	14.317
	Role physical	20.32	62.91	46.776	13.296
	Bodily pain	16.68	63.90	47.595	14.067
	General health	18.87	64.61	48.499	12.820
	Vitality	34.61	67.88	54.790	10.324
	Social Functioning	16.18	65.70	52.826	12.319
	Role emotional	22.53	68.81	42.937	14.755
	Mental health	40.16	65.73	54.038	9.553
	Physical Component state	17.43	64.84	48.639	15.795
	Mental Component state	29.21	75.48	51.449	10.025
EuroQoL 5D		0.28	1.00	0.815	0.193
EuroQoL VAS		28.00	97.00	78.290	20.966
N		424			

The minimum and maximum values of the APFQ-Sp questionnaire were reached by 1.65% and 4.48% of the participants, respectively, completing the questionnaire in an average time of 18 min. The general internal consistency of the questionnaire was 0.972 and the intraclass correlation coefficient ranged between ICC: 0.596 – 0.960) (Table 3). On the other hand, the SEM and MDC90 values were 0.04 and 0.009, respectively.

**Table 3:** Reliability and Response to the item (ICC) of the APFQ-Sp.

	Urinary tract section	Defecatory section	Prolapse section	Sexual activity section	Total
Cronbach’s Alpha	0.935	0.919	0.885	0.828	0.972
ICC (item responses)	0.596–0.960				

**Figure 2:**
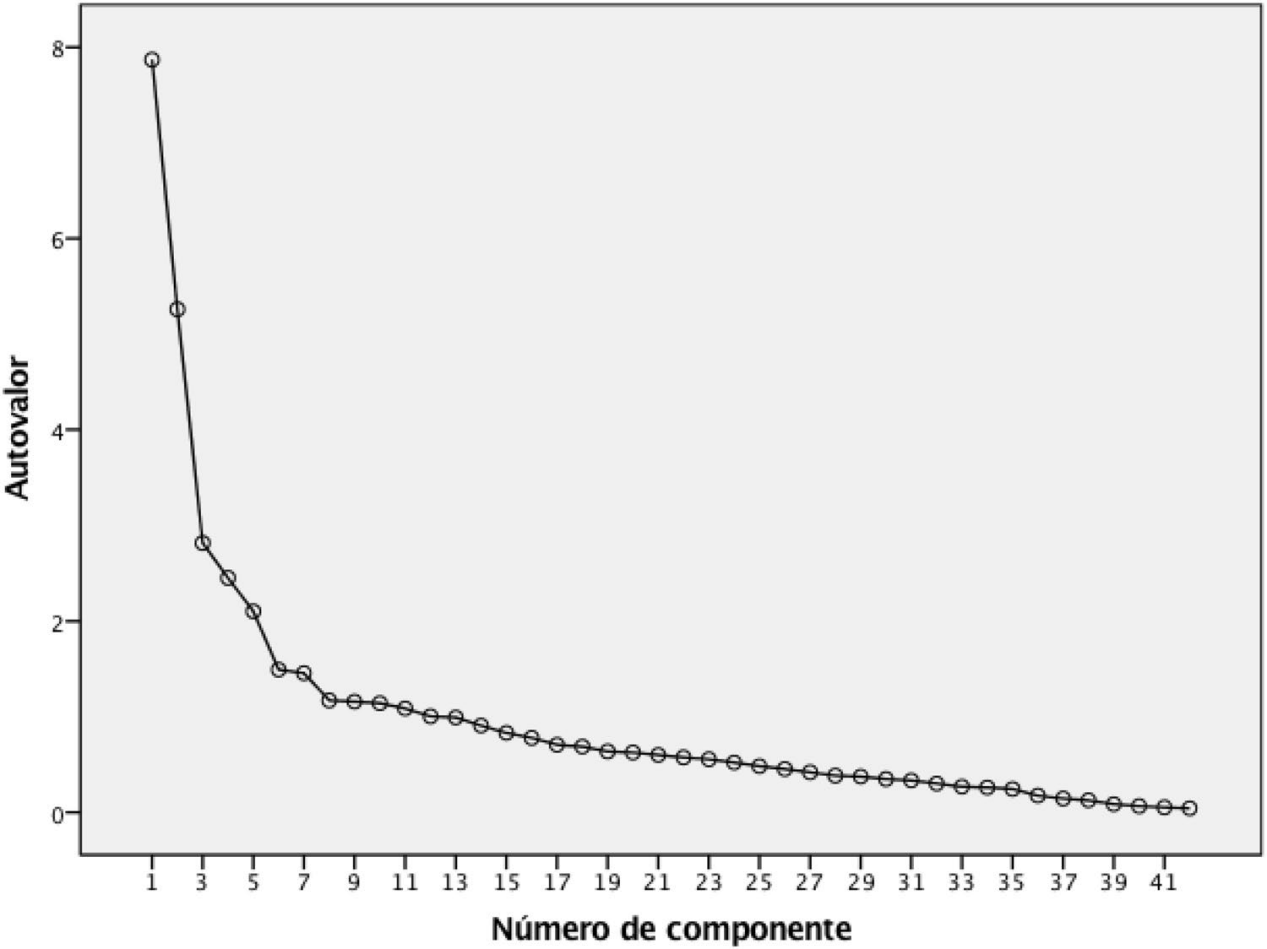
Sedimentation graph.

In construct validity, the maximum likelihood extraction method presented a value of 0.858 in the Kaiser–Meyer–Olkin test, with a significant value in the Bartlett sphericity test (p < 0.001) and a Chi-square value of 10,432.61 in the Bartlett sphericity test and in the Kaiser–Meyer–Olkin test (0.833). The APFQ-Sp presents a solution of two factors, since they are the only two factors that explain more than 10% of the variance each (18.737% and 12.521%, respectively); however, there is a wide distribution of the variance explained in the APFQ-Sp, since up to question 14 the explained variance exceeds 2% (Table 4). Figure 2 shows the sedimentation graph, while Table 5 shows the load of each of the questions in the two factors that met the established criteria; specifically, in factor 1, questions 33–41 exceeded 0.5 of load factor, while in the second factor, questions 4, 5, 6, 14, 15, 29 and 32 exceeded this value.

**Table 4:** Total variance explained by the four factors extracted according to the structure of the original APFQ.

Component	Initial eigenvalues			Sums of extraction of charges squared		
	Total	Variance %	% accumulated	Total	Variance %	% accumulated
1	7.870	18.737	18.737	7.870	18.737	18.737
2	5.259	12.521	31.259	5.259	12.521	31.259
3	2.818	6.708	37.967	2.818	6.708	37.967
4	2.453	5.840	43.807	2.453	5.840	43.807
5	2.104	5.008	48.816			
42	0.043	0.103	100.000

**Table 5:** Load factor of the 4 factors extracted from an exploratory factor analysis.

	Component			
	1	2	3	4
1. How many times do you pass urine in the day?	0.095	0.228	−0.038	−0.195
2. How many times do you get up at night to pass urine?	0.186	0.268	0.138	−0.291
3. Do you wet the bed before you wake up?	0.029	0.201	−0.102	−0.002
4. Do you need to rush/hurry to pass urine when you get the urge?	0.204	**0.518**	−0.114	−0.302
5. Does urine leak when you rush/hurry to the toilet/Can you make it in time?	0.191	**0.509**	−0.178	−0.206
6. Do you leak with coughing, sneezing, laughing, exercising?	0.165	**0.601**	−0.136	−0.058
7. Is your urinary stream/flow weak/prolonged/slow?	0.188	0.433	−0.029	−0.092
8. Do you have a feeling of incomplete bladder emptying?	0.215	0.465	0.027	−0.209
9. Do you need to strain to empty your bladder?	0.129	0.393	0.057	−0.159
10. Do you have to wear pads?	0.057	0.483	−0.130	−0.183
11. Do you limit your fluid intake to decrease leakage?	0.186	0.412	−0.086	−0.308
12. Do have frequent bladder infections?	−0.019	0.194	0.150	−0.139
13. Do you have pain in your bladder/urethra when you empty your bladder?	0.035	0.307	0.029	−0.309
14. Does urine leakage affect your routine activities (recreation, shopping etc.)	0.222	**0.519**	−0.165	−0.151
15. How much of a bother is your bladder problem to you?	0.164	**0.692**	−0.150	−0.143
16. How often do you usually open your bowels?	−0.020	0.154	0.421	0.324
17. How is the consistency of your usual stool?	−0.018	−0.011	0.042	−0.023
18. Do you have to strain a lot to empty your bowels?	0.120	0.197	**0.653**	0.342
19. Do you use laxatives to empty your bowels?	0.000	0.110	0.395	0.181
20. Do you feel constipated?	0.129	0.217	**0.715**	0.267
21. When you get wind/flatus, can you control it or does wind leak?	0.186	0.316	0.256	−0.189
22. Do you get an overwhelming sense of urgency to empty bowels?	0.146	0.269	0.187	−0.309
23. Do you leak watery stool when you don’t mean to?	0.205	0.355	0.113	−0.110
24. Do you leak normal stool when you don’t mean to?	0.130	0.295	0.114	−0.235
25. Do have the feeling of incomplete bowel emptying?	0.248	0.299	0.508	0.082
26. Do you use finger pressure to help empty your bowel?	0.143	0.072	0.417	0.188
27. How much of a bother is your bowel problem to you?	0.200	0.402	**0.578**	0.044
28. Do you get a sensation of tissue protrusion in your vagina/lump/ bulging?	0.135	0.479	−0.376	**0.604**
29. Do you experience vag. pressure/ heaviness/dragging sensation?	0.162	**0.530**	−0.200	0.396
30. Do you have to push back your prolapse in order to void?	0.140	0.405	−0.381	0.551
31. Do you have to push back your prolapse to empty your bowels?	0.082	0.272	−0.098	0.281
32. How much of a bother is the prolapse to you?	0.115	**0.519**	−0.311	**0.569**
33. Are you sexually active? *If not sexually active, please answer questions 34 and 42 only*	−**0.771**	0.210	0.103	−0.081
34. If NOT, why not:	−**0.861**	0.306	0.010	0.023
35. Do you have sufficient lubrication during intercourse?	**0.916**	−0.278	−0.024	0.002
36. During intercourse vaginal sensation is:	**0.932**	−0.212	−0.032	0.006
37. Do you feel that your vagina is too loose or lax?	**0.906**	−0.121	−0.082	−0.032
38. Do you feel that your vagina is too tight?	**0.911**	−0.273	−0.005	0.006
39. Do you experience pain with intercourse:	**0.934**	−0.228	−0.001	0.002
40. Where does the pain occur	**0.775**	−0.124	0.007	0.024
41. Do you leak urine during sex?	**0.924**	−0.256	−0.054	−0.010
42. How much of a bother are these sexual issues to you?	0.367	0.320	0.019	0.156

Significant values are in bold.

When analysing the correlation between the total value of the APFQ-Sp and its sub-scales, it is observed that the levels of correlation oscillate between r = 0.103 (Defecation Section – Sexual Activity Section) and r = 0.752 (Total APFQ – Sexual Activity Section) (Table 6). In addition, in the calculation of the criterion validity (Table 6), significant correlation values were obtained, with a range of r = 0.285–0.776 in FSFI, r = 0.103–0.778 in KHQ, r = 0.122–0.872 in SF-12 and r = 0.384–0.817 in EuroQoL. The rest of the correlation values are presented in Table 6.

**Table 6:** Correlation matrix between the APFQ-Sp, its different dimensions and the IFSF, KHQ, SF-12 and EuroQoL-5D questionnaires.

		Total	Urinary tract section	Defecatory section	Prolapse section	Sexual activity section
APFQ	Total	1	Good	Moderate	Moderate	Good
	Urinary tract section	0.649**	1	Poor	Poor	Poor
	Defecatory section	0.532**	0.290**	1	Poor	Poor
	Prolapse section	0.550**	0.330**	0.122*	1	Poor
	Sexual activity section	0.752**	0.207**	0.103*	0.192**	1
FSFI	FSFI_Total puntuation	0.776**	0.485**	0.421**	0.404**	0.601**
	FSFI orgasm domain	0.605**	0.390**	0.346**	0.319**	0.450**
	FSFI satisfaction domain	0.523**	0.335**	0.285**	0.320**	0.376**
	FSFI_Pain domain	0.575**	0.332**	0.289**	0.375**	0.438**
KHQ	Q1. 1. How would you describe your general health?	0.616**	0.425**	0.328**	0.329**	0.455**
	Q2. 2. To what extent do you think your urinary problems affect your life?	0.572**	0.535**	0.259**	0.342**	0.351**
	KHQ Part 1	0.778**	0.621**	0.387**	0.437**	0.531**
	KHQ role limitation	0.516**	0.400**	0.265**	0.281**	0.359**
	KHQ physical limitation	0.478**	0.523**	0.179**	0.283**	0.272**
	KHQ social limitation	0.538**	0.402**	0.307**	0.323**	0.349**
	KHQ personal relationships	0.426**	0.334**	0.103*	0.227**	0.361**
	KHQ emotions	0.489**	0.491**	0.087	0.441**	0.287**
	KHQ sleep energy	0.326**	0.358**	0.156**	0.249**	0.140**
	KHQ severity	0.470**	0.598**	0.232**	0.263**	0.197**
	KHQ Part 2	0.616**	0.701**	0.207**	0.386**	0.339**
	KHQ Part 3	0.548**	0.650**	0.269**	0.293**	0.262**
SF-12	Physical function	0.684**	0.506**	0.367**	0.338**	0.496**
	Role physical	0.702**	0.476**	0.394**	0.350**	0.522**
	Bodily pain	0.769**	0.510**	0.413**	0.402**	0.580**
	General health	0.728**	0.492**	0.438**	0.274**	0.569**
	Vitality	0.082	0.098*	0.030	0.039	0.047
	Social functioning	0.708**	0.442**	0.380**	0.448**	0.512**
	Role emotional	0.217**	0.143**	0.090	0.122*	0.175**
	Mental health	0.159**	0.142**	0.058	0.043	0.135**
	Physical component state	0.786**	0.538**	0.422**	0.389**	0.595**
	Mental component state	0.872**	0.622**	0.546**	0.418**	0.606**
EuroQoL_5D		0.817**	0.537**	0.405**	0.443**	0.630**
EuroQoL VAS		0.720**	0.435**	0.391**	0.384**	0.561**

## Discussion

This study aimed to carry out a cross-cultural adaptation and a validation study of the tool for the assessment and monitoring of pelvic floor dysfunction APFQ into Spanish. The translation and cross-cultural adaptation of the APFQ-Sp was carried out following the recommendations of the literature, which ensures the conceptual equivalence between the translated version and the original version and it is essential to facilitate the use of the APFQ-Sp among Spanish speakers, while favouring the comparison of potential results with versions of the APFQ published in other languages. Based on the cross-cultural adaptation process carried out, the APFQ-Sp proved to be an understandable and easy-to-use tool.

### Translation of the APFQ to APFQ-SP and cross-cultural adaptation

In addition to the original version of the APFQ^18^, other versions of the APFQ have been published, such as the Turkish^22^, Chinese^20,40^, Arabic^21^, Serbian^19^ and German^41^ versions. Both the translation from the original version to the Spanish version and the back-translation were carried out by native translators to guarantee the equivalence of the terms between both versions, which facilitates its use among researchers and clinician Spanish speakers.

### Construct validity

To assess the construct validity, the structure of the original version of the APFQ was taken into account, where 4 factors are identified. In this sense, two factors explain a level of variance greater than 10% and, in addition, in the scree plot they show a change in proportion in the level of explained variance. In this sense, if all the criteria that are usually considered for factor extraction had been taken into account (> 10% of the variance, eigenvalue > 1.0, and scree plot inflection point), two factors of APFQ-Sp would have been extracted. In this sense, the only version whose construct validity has been analysed is the Arabic version, which shows values of KMO = 0.806 and Bartlett sphericity test = 4150.46. It would be interesting to carry out studies to analyse the construct validity of the rest of the versions and determine whether they behave in a similar way as the versions that have performed such analysis (Spanish and Arabic).

### Internal consistency and test–retest

The internal consistency in the APFQ-Sp showed a Cronbach's α of 0.972, and, in the sub-scales, it ranged between Cronbach's α = 0.828 (sexual activity section) and 0.935 (urinary tract section) (Table 3). These values are slightly higher than those observed in the Arabic (0.877)^21^, Chinese (0.83–0.89)^20,40^, Serbian (0.822–0.846)^19^, Turkish (0.733–0.858)^22^ and original version (0.74–1.00)^18^, although all except one dimension from the Turkish version and one from the original version are considered to have excellent internal consistency^36^.

When the test–retest values are compared, since the APFQ-Sp has ICC values that range between 0.596 and 0.960 (Table 3), it is observed that they are consistent with the Arabic version^21^, which presents ICC values: 0.500–0.833. However, these values are slightly lower than those observed in the Serbian (ICC: 0.896–0.944)^19^, Turkish (ICC: 0.876–0.954)^22^, and original version (ICC: 0.74–1.0)^18^, and greater than some dimensions of the Chinese version (ICC: 0.22–0.88)^20,40^.

### Criterion validity

The values observed in the criterion validity when comparing the APFQ-Sp with the rest of the questionnaires (FSFI, KHQ, SF-12, EuroQol_5D, EuroQol_VAS) and their different subdimensions show that the total value of the questionnaire tends to correlate better with all the reference questionnaires in comparison with the dimensions of the APFQ-Sp. In this sense, when the results are compared with other versions, it is observed that, with the exception of the original version, the APFQ-Sp is the only one that evaluated this psychometric aspect. The original version performs a convergence analysis with the short version of the Urogenital Distress Inventory (SUDI), showing correlation levels of r = 0.80, while the level of correlations between pelvic organ prolapse and prolapse symptoms quantification measurements (measured in 106 patients) showed a range of r = 0.25–0.68. In this sense, it is observed that the APFQ complements very well with other questionnaires or diagnostic instruments for patients with pelvic floor problems, although it would be interesting to know the level of correlation of the other versions to have a much more complete perspective.

### Implications for future research

There is a need for developing valid and reliable instruments to measure pelvic floor dysfunctions in order to provide accurate and consistent measurements over time^42,43^. These instruments must be concise, valid, reliable, evidence-based and developed using best practices^42,43^. In this context, the APFQ is a measure that was proposed to evaluate pelvic floor dysfunctions in women^18^. The APFQ was developed based on the most valid and reliable questions to focus on the main pelvic floor dysfunctions in women through a systematic review of the literature to identify measures with the best psychometric properties. This study provides evidence for the validity of the APFQ-Sp. In this sense, the APFQ-Sp is a concise, valid, reliable and evidence-based document and, at the same time, it is an instrument developed using best practices. Therefore, the APFQ-Sp is a measure that can be recommended for the assessment of pelvic floor dysfunction in Spanish female runners.

The current study demonstrates that the APFQ-Sp is a valid measure to assess pelvic floor dysfunction in the Spanish population, which allows researchers and clinicians to use this tool within both clinical and research settings. In this sense, research on the pelvic floor is an area of special interest, since it has implications for the development of interventions for both the prevention and treatment of pelvic floor dysfunctions in the female population. In addition, future research should study the APFQ in different clinical populations, such as cancer related to the pelvic floor, or analyse some psychometric variables that have not been taken into account in this validation study, and that are linked to longitudinal studies, such as the sensitivity to change.

### Strengths and weaknesses

This study presents some strengths that show the appropriateness of its execution. The main strength is that it allows the APFQ to be adapted into Spanish, the second most widely spoken language in the world and one of the five official languages of the UN. On the one hand, this cross-cultural adaptation and validation benefits the entire Spanish-speaking clinical population, and, on the other hand, the results obtained with this instrument can be compared with other population groups that have used other versions, such as the original (English)^18^, Turkish^22^, Arabic^21^, Serbian^19^ and Chinese^20,40^ versions. In addition, the cross-cultural adaptation and the subsequent validation study were carried out respecting the minimum number of subjects recommended in the literature^38^. In this sense, there would be 420 minimum necessary subjects, and it was carried out with 424 participants.

However, there are some weaknesses that must be taken into account when interpreting the results of this study. Specifically, this study did not perform the psychometric analysis of longitudinal variables, such as error scores, responsiveness or sensitivity to change. In this sense, future studies should be designed and executed in order to assess these psychometric variables in the APFQ-SP.

The cross-cultural adaptation and validation of the APFQ-SP has been carried out in a specific group of women with a higher level of incidence of PFDs. However, there are other population groups that also have a higher incidence of PFDs, such as multiparous women or women over 65 years of age, so future studies should be designed to validate the APFQ in these specific population groups.

## Conclusions

The Spanish version of the APFQ is a valid and reliable measure that can be used clinically to assess pelvic floor dysfunctions in the Spanish female population. This instrument is complete and includes different dimensions on the most relevant aspects and symptoms of female pelvic floor dysfunctions, allowing its use by both researchers and clinical professionals, who speak Spanish, for the evaluation and follow-up of patients with pelvic floor dysfunctions.

## Supplementary Information

Supplementary Information.

## Data Availability

All data generated or analysed during this study are included in this published article [and its supplementary information files].
