# Supplementary material for: Spanish cross-cultural adaptation and validation of the Australian Pelvic Floor Questionnaire in running women
Source: Sci Rep. 2022 May 18;12:8325. doi: 10.1038/s41598-022-12043-5 (PMC9117665; doi:10.1038/s41598-022-12043-5)
Supplement: Supplementary file 1 — Supplementary Information. [file 41598_2022_12043_MOESM1_ESM.pdf]

Supplementary File 1. Spanish version of Australian Pelvic Floor Questionnaire (APFQ-Sp)

**Sección tracto urinario:**	**P 1 – 15**	**Puntuación____/ 45 = ____**
**1.Frecuencia miccional**<br>¿Cuántas veces orina al día?<br><br>(0) Hasta 7 veces<br>(1) Entre 8 -10 veces<br>(2) Entre 11 – 15 veces<br>(3) Más de 15 veces	**2.Nocturia**<br>¿Cuántas veces se levanta por la noche para orinar?<br><br>(0) 0 – 1 vez<br>(1) 2 veces<br>(2) 3 veces<br>(3) Más de 3 veces	**3.Enuresis nocturna**<br>¿Cuándo se despierta tiene la cama mojada?<br><br>(0) Nunca<br>(1) Ocasionalmente, menos de 1 vez/semana<br>(2) Frecuentemente, una o más veces/semana<br>(3) Siempre, cada noche
**4.Urgencia**<br>¿Necesita ir con rapidez a orinar cuando tiene ganas?<br><br>(0) Nunca<br>(1) Ocasionalmente, menos de 1 vez/semana<br>(2) Frecuentemente, una o más veces/semana<br>(3) A diario	**5.Incontinencia de urgencia**<br>¿Tiene pérdidas de orina cuando le urge ir al baño?<br><br>(0) Nunca<br>(1) Ocasionalmente, menos de 1 vez/semana<br>(2) Frecuentemente, una o más veces/semana<br>(3) A diario	**6.Incontinencia de esfuerzo**<br>¿Tiene pérdidas de orina tosiendo, estornudando, riendo, haciendo ejercicio?<br><br>(0) Nunca<br>(1) Ocasionalmente, menos de 1 vez/semana<br>(2) Frecuentemente, una o más veces/semana<br>(3) A diario
**7.Chorro débil**<br>¿Es su chorro de orina débil/prolongado/lento?<br><br>(0) Nunca<br>(1) Ocasionalmente, menos de 1 vez/semana<br>(2) Frecuentemente, una o más veces/semana<br>(3) A diario	**8.Vaciado incompleto vesical**<br>¿Tiene sensación de vaciado incompleto de la vejiga?<br><br>(0) Nunca<br>(1) Ocasionalmente, menos de 1 vez/semana<br>(2) Frecuentemente, una o más veces/semana<br>(3) A diario	**9.Esfuerzo evacuatorio**<br>¿Tiene que forzar para vaciar su vejiga?<br><br>(0) Nunca<br>(1) Ocasionalmente, menos de 1 vez/semana<br>(2) Frecuentemente, una o más veces/semana<br>(3) A diario
**10.Uso de compresa**<br>¿Necesita usar compresas?<br><br>(0) No, nunca<br>(1) Por precaución<br>(2) En el ejercicio/durante los resfriados<br>(3) A diario	**11.Ingesta reducida de líquidos**<br>¿Limita su ingesta de líquidos para disminuir las pérdidas?<br><br>(0) Nunca<br>(1) Antes de salir de casa<br>(2) Moderadamente<br>(3) A diario	**12.ITU (infección del tracto urinario) recurrente**<br>¿Tiene infecciones de orina frecuentes?<br><br>(0) No<br>(1) 1 - 3 infecciones/año<br>(2) 4 - 12 infecciones/año<br>(3) Más de 1 infección/mes
**13.Disuria**<br>¿Tiene dolor cuando orina?<br>U **otros síntomas** como hematuria, dolor, etc.	**14.Impacto en la vida social**<br>¿Las pérdidas de orina afectan a sus actividades	**15.¿Cuánto le preocupa** su problema urinario?<br><br>(0) Sin problema

(0) Nunca<br>(1) Ocasionalmente, menos de 1 vez/semana<br>(2) Frecuentemente, una o más veces/semana<br>(3) A diario	cotidianas (recreacional, compras, etc.)?<br><br>(0) De ningún modo<br>(1) Ligeramente<br>(2) Moderadamente<br>(3) Mucho	(1) Ligeramente<br>(2) Moderadamente<br>(3) Mucho
**Sección tracto intestinal**	**P 16-27**	**Puntuación____/34 = ____**
**16.Frecuencia defecatoria**<br>¿Con que frecuencia defeca?<br><br>(2) Menos de 1 vez/semana<br>(1) Menos de 1 vez cada 3 días<br>(0) Menos de 3 veces/semana o a diario<br>(0) Más de 1/día	**17.Consistencia de las heces**<br>¿Cómo es la consistencia habitual de sus heces?<br><br>(0) Blandas<br>(0) Firmes<br>(1) Duras/bolitas<br>(2) Acuosas<br>(1) Variable	**18.Esfuerzo de defecación**<br>¿Tiene que hacer mucho esfuerzo para vaciar defecar?<br><br>(0) Nunca<br>(1) Ocasionalmente, menos de 1 vez/semana<br>(2) Frecuentemente, una o más veces/semana<br>(3) A diario
**19.Uso de laxantes**<br>¿Usa laxantes para defecar?<br><br>(0) Nunca<br>(1) Ocasionalmente, menos de 1 vez/semana<br>(2) Frecuentemente, una o más veces/semana<br>(3) A diario	**20.¿Se siente estreñida?**<br><br>(0) Nunca<br>(1) Ocasionalmente, menos de 1 vez/semana<br>(2) Frecuentemente, una o más veces/semana<br>(3) A diario	**21.Incontinencia de gases**<br>Cuando tiene gases, ¿tiene fugas?<br><br>(0) Nunca<br>(1) Ocasionalmente, menos de 1 vez/semana<br>(2) Frecuentemente, una o más veces/semana<br>(3) A diario
**22.Urgencia fecal/defecatoria**<br>¿Tiene una sensación imperiosa de urgencia para defecar?<br><br>(0) Nunca<br>(1) Ocasionalmente, menos de 1 vez/semana<br>(2) Frecuentemente, una o más veces/semana<br>(3) A diario	**23.Incontinencia fecal con diarrea**<br>¿Tiene fugas de heces acuosas?<br><br>(0) Nunca<br>(1) Ocasionalmente, menos de 1 vez/semana<br>(2) Frecuentemente, una o más veces/semana<br>(3) A diario	**24.Incontinencia fecal con heces normales**<br>¿Tiene fugas de heces de consistencia normal?<br><br>(0) Nunca<br>(1) Ocasionalmente, menos de 1 vez/semana<br>(2) Frecuentemente, una o más veces/semana<br>(3) A diario
**25.Vaciado intestinal incompleto**<br>¿Tiene la sensación de un vaciado intestinal incompleto? U o**tros síntomas** como dolor, secreción mucosa, prolapso rectal, etc.<br><br>(0) Nunca<br>(1) Ocasionalmente, menos de 1 vez/semana	**26.Obstrucción defecatoria**<br>¿Usa la presión de sus dedos para ayudarse a defecar?<br><br>(0) Nunca<br>(1) Ocasionalmente - menos de 1 vez/semana<br>(2) Frecuentemente, una o más veces/semana<br>(3) A diario	**27 ¿Cuánto le preocupa o molesta** su problema intestinal?<br><br>(0) Sin problema<br>(1) Ligeramente<br>(2) Moderadamente<br>(3) Mucho

(2) Frecuentemente, una o más veces/semana<br>(3) A diario		
**Sección prolapso**	**P 28 – 32**	**Puntuación ___/ 15 = ___**
**28.Sensación de prolapso** ¿Nota algún bulto a nivel de su orificio vaginal?<br><br>(0) Nunca<br>(1) Ocasionalmente, menos de 1 vez/semana<br>(2) Frecuentemente, una o más veces/semana<br>(3) A diario	**29.Presión vaginal o pesadez** ¿Experimenta presión vaginal o pesadez?<br><br>(0) Nunca<br>(1) Ocasionalmente, menos de 1 vez/semana<br>(2) Frecuentemente, una o más veces/semana<br>(3) A diario	**30.Reducción del prolapso** ¿Necesita recolocar el prolapso?<br><br>(0) Nunca<br>(1) Ocasionalmente, menos de 1 vez/semana<br>(2) Frecuentemente, una o más veces/semana<br>(3) A diario
**31.Reducción del prolapso para defecar**<br>¿Tiene que recolocar su prolapso para defecar? U **otros síntomas** como problemas para sentarse/caminar, dolor, sangrado vaginal, etc.<br><br>(0) Nunca<br>(1) Ocasionalmente, menos de 1 vez/semana<br>(2) Frecuentemente, una o más veces/semana<br>(3) A diario	**32.¿Cuánto le preocupa** su prolapso?<br><br>(0) Sin problema<br>(1) Ligeramente<br>(2) Moderadamente<br>(3) Mucho	
**Sección función sexual**	**P 33 – 42**	**Puntuación ___/ 21= ___**
**33.Actividad sexual**<br>¿Es usted sexualmente activa?<br>**Si NO es sexualmente activa, por favor responda solo las preguntas 34 y 42**<br><br>___No<br>___Menos de 1 vez/semana<br>___Uno o más veces/semana<br>___Casi todos los días/a diario<br><br>Esta pregunta NO puntúa	**34.Si NO, porque:**<br><br>___Sin pareja (0)<br>___Pareja incapaz (0)<br><br>___Sequedad vaginal<br>___Demasiado doloroso<br>___Vergüenza (prolapso/incontinencia)<br><br>___Otros (0) ✧<br><br>✧ 18	**35.Suficiente lubricación** ¿Tiene suficiente lubricación durante el coito?<br><br>(0) Sí<br>(1) No
**36.Durante el coito la sensación vaginal es:**<br><br>(0) Normal/placentera<br>(1) Mínima<br>(1) Dolorosa<br>(3) Ninguna	**37.Laxitud vaginal** ¿Tiene la sensación de que su vagina es demasiado floja?<br><br>(0) Nunca<br>(1) Ocasionalmente<br>(2) Frecuentemente	**38.Opresión vaginal/vaginismo** ¿Siente que su vagina está demasiado prieta?<br><br>(0) Nunca<br>(1) Ocasionalmente

	(3) Siempre	(2) Frecuentemente<br>(3) Siempre
**39.Dispareunia**<br>¿Siente dolor durante el coito?<br><br>(0) Nunca<br>(1) Ocasionalmente<br>(2) Frecuentemente<br>(3) Siempre	**40.Localización de la dispareunia**<br>¿Dónde aparece el dolor? U **otros síntomas como** aires vaginales o incontinencia fecal, vaginismo, etc.<br><br>(0) No hay dolor<br>(1) En la entrada de la vagina<br>(1) En el fondo/en la pelvis<br>(2) Ambos	**41.Incontinencia coital**<br>¿Tiene pérdidas de orina durante el sexo?<br><br>(0) Nunca<br>(1) Ocasionalmente<br>(2) Frecuentemente<br>(3) Siempre
**42.¿Cuánto le preocupan** estos problemas sexuales?<br><br>(0) No hay problema<br>(1) Problema pequeño<br>(2) Problema moderado<br>(3) Gran problema		
**Puntuación TOTAL Disfunción del suelo pélvico _______________ /40**		

Como puntúa el Australian Pelvic Floor Questionnaire (APFQ):

- La puntuación del APFQ se puede realizar por secciones, para poder tener una visión de la afectación urinaria, coloproctológica y sexual de las pacientes. Así como obtener una puntuación final en la cual se encuentran todas las secciones aunadas.
- PUNTUACIÓN POR SECCIONES:
  - Sección tracto urinario: (suma de las puntuaciones de las preguntas 1 a 15/45)*10
  - Sección tracto intestinal: (suma de las puntuaciones de las preguntas 16 a 27/34)*10
  - Sección prolapso: (suma de las puntuaciones de las preguntas 28 a 32/15)*10
  - Sección función sexual:
    - Si NO es sexualmente activa: solo responder la pregunta 34 (puntuación 0 o 18) y 42
    - Si es sexualmente activa: responder de la pregunta 35 a la 42
    - En ambos casos: (suma de las puntuaciones de las preguntas correspondientes/21)*10
- PUNTUACIÓN TOTAL: (suma de la puntuación de las 4 secciones/40)*10

- La puntuación, sea de las secciones o la final, será entre 0 y 10. Siendo 0 la puntuación que indica que la mujer no tiene ninguna problemática a nivel del suelo pélvico, y 10, la puntuación que nos indica la máxima disfunción a nivel perineal.
